# Classification of multivariate data with a spiking neural network on neuromorphic hardware

**DOI:** 10.1186/1471-2202-14-S1-P290

**Published:** 2013-07-08

**Authors:** Michael Schmuker, Thomas Pfeil, Martin P Nawrot

**Affiliations:** 1 Theoretical Neuroscience, Institute of Biology, Freie Universität Berlin, Berlin, 14195, Germany; 2 Bernstein Center for Computational Neuroscience Berlin, Berlin, 10115, Germany; 3 Kirchhoff-Institute for Physics, Heidelberg University, Heidelberg, 69120, Germany

## 

Progress in the field of computational systems neuroscience has uncovered a number of computational principles employed by nervous systems. At the same time neuromorphic hardware systems have evolved to a state where fast *in silico *implementations of complex neural networks are feasible. The current challenge is to identify and implement functional neural networks that enable neuromorphic computing to solve real world problems. Here, we present a generic spiking neural network for the supervised classification of multivariate data, a common problem in signal and data analysis. The network architecture was inspired by the data processing scheme of the olfactory system [1]. It has a three stage architecture. In the first stage, real-valued multivariate data is encoded into a bounded, positive firing-rate representation. The second stage removes correlation between input channels through lateral inhibition. Supervised training affects synaptic weights in the third stage, where classification of input patterns is performed.

We implemented and tested our network on the *Spikey *neuromorphic hardware system [2]. Our network performed on the same level as a Naïve Bayes classifier on several benchmark data sets. Our classifier network is an important proof-of-principle for a bio-inspired functional spiking network implemented on neuromorphic hardware performing a real-world computing task.

This study was funded by the DFG (SCHM2474/1-1, MS), the BMBF (01GQ1001D, MS and MN, 01GQ0941, MN) and the European Union (243914, TP).
